# Induction of resistance to *Myzus persicae-nicotianae* in *Cucumber mosaic virus* infected tobacco plants using silencing of CMV-2b gene

**DOI:** 10.1038/s41598-022-08202-3

**Published:** 2022-03-08

**Authors:** Kazhal Karimi, Amin Sadeghi, Mostafa Maroufpoor, Abdolbaset Azizi

**Affiliations:** Department of Plant Protection, University of Kurdistan, Sanandaj, Iran

**Keywords:** Biotechnology, Microbiology, Plant sciences

## Abstract

Aphids such as tobacco aphid *Myzus persicae-nicotianae,* are among the most important plant viral vectors and plant viruses encode genes to interact with their vectors. *Cucumber mosaic virus* (CMV) encodes 2b protein as a suppressor of plant immune and it plays a vital role in CMV accumulation and susceptibility to aphid vectors. In this study, the resistance of tobacco plants (*Nicotiana tabacum*) to *M. p. nicotianae* was evaluated by silencing of *2b* in CMV-infected plants. However, the *pFGC-C.h* silencing gene construct was transiently expressed using *Agrobacterium tumefacience,* LBA 4404 in tobacco leaves, and four days later, the plants were mechanically inoculated by CMV (Kurdistan isolate), and then, 15 days post-inoculation 1 nonviruliferous aphid was placed on each leaf for evaluation of resistance to *M. p. nicotianae*. To evaluate the tobacco plants resistance and susceptibility to *M. p. nicotianae*, the number of aphids existent per tobacco leaf, life table and, demographic parameters were recorded and used as a comparison indicator. The obtained results were analyzed using the age-stage, two-sex life table. The highest number of aphids was recorded on the control CMV-infected plants, while the lowest number on CMV infected leaves expressing CMV-2b silencing construct (*pFGC-C.h*). The obtained data revealed the lowest rate for all of intrinsic rate of natural increase (r_m_) (0.246/day), the rate of reproduction (r0) (17.04 females/generation), and finite rate of increase (λ) (1.279/day), on the *pFGC-C.h* treatment. The maximum generation time (T) (11.834 days) was observed on (V) treatment. However, the collected data revealed induction of resistance to tobacco aphids by silencing of CMV-2b in CMV infected plants.

## Introduction

*Cucumber mosaic virus* (CMV) is one of the most important worldwide plant pathogens with a broad host range for more than 1300 plant species^1^ and it causes quantitative and qualitative damage to agricultural products^2^. CMV is a type species of the *Cucumovirus* genus (Bromoviridae) and is transmitted nonpersistent by different aphid species, especially *Myzus persicae*^3^. Other transmission ways for CMV are mechanically such as agricultural tools and moving between healthy and infected plants, seeds, and vegetative propagating^4,5^. Because *M. persicae* is a worldwide herbivore with a wide plant host range^6–8^, plays an important role in virus transmission.

Control of plant viruses like CMV with a broad host range and has more than 80 aphid vector species from 33 genera, is a big challenge in agriculture^7,8^.

Viruses alter plant-insects and virus-vectors interactions, increase virus transmission efficiency, and may it favor vectors spreading in the fields^7,9–12^. Fny strain of CMV (Fny-CMV) induces aphid attraction to the infected plants by changes in the emission of plant volatile compounds^13^, increases aphids density on the CMV infected plants, and also arouse birth of winged aphids to promote virus transmission in more long-distance at the landscape scale^10^.

However, it was estimated that 50% of insect-borne viruses are transmitted by aphids^14^. Because of some unique features, aphids becomes a suitable vector for plant viruses such as polyphagous insect, quickly producing a large number of progeny, and the ability to pierce the plant wall^15^.

RNAi is an evolutionarily conserved process in eukaryotic cells that plays an important role in defense against invasive nucleic acids, such as transposable elements and viruses using small interfering RNAs to suppress homologous sequences; and also it is an important mechanism for regulating endogenous gene expression^16–18^.

CMV-2b is a strong suppressor of plant RNA silencing by direct blocking of RISC activity, and interferes with target RNA cleavage by inhibition of miRNA and siRNA and, plays an important role for cucumoviruses in breaking the host resistance^19^. Plants expressing 2b show developmental abnormality and the severity of this abnormality correlated with the severity of the perturbation in the miRNA pathways^19^.

2b not only inhibits RNA silencing against viruses but also suppresses genes in the jasmonic acid pathway, a key molecule in systemic signaling of resistance against plant necrotrophic pathogens and insects^20–22^. Moreover, tobacco infected with a CMVΔ2b which contains defective *2b,* showed induction of strong resistance against *M. persicae* and indicated that 2b protein inhibits plants to induce antibiosis against aphids^20,23^.

CMV-2b induces gene expression in the pathway of SA and interferes with the expression of genes related to JA by induction of NPR1 and PR1. However, it plays an important role in the SA and JA antagonistic crosstalk^24–26^.

However, suppressing of jasmonic acid signaling pathway by 2b affects the behavior of aphids and leads to the attraction of aphids to the virus-infected plant^13^.

Based on the combined knowledge about the suppression of JA resistance pathway by the CMV-2b, and the strong role of the JA pathways in resistance to pests, and also the resistance to aphids by a mutation in CMV-, in the present study, an attempt was made to investigate the induction of resistance to *M. persicae* using silencing of CMV-2b in the CMV infected plants. However, the gene construct containing 2b hairpin structure has been transiently expressed in CMV infected tobacco plants using agrobacterium infiltration, and the resistance to *M. persicae* has been analyzed.

## Results

In this research, the effect of 2b silencing on the resistance to *M. persicae* was conducted using transient expression of 2b silencing gene construct and after that, the aphid demographic parameters were evaluated. To confirm the healthy and CMV infected, plants the ELISA was conducted. Healthy and CMV infected plants used in this research were confirmed using Indirect Enzyme-linked immunosorbent assay (ELISA) (Fig. S2) and RT-PCR (Fig. S3), and the results confirmed the infection of mechanically infected plants and also revealed that the aphid population used in this research are not CMV infected before. PCR result showed a 519 bp CMV band as expected in inoculated plants (Fig. S3). ELISA results showed lower virus concentration in plants expressing *pFGC-C.h* (CMV-2b.h) construct, compared to the plants expressing empty vector (*pFGC*5941) (Fig. S2), Therefore, it revealed that expression of the *pFGC-C.h* (CMV-2b.h) gene construct causes a reduction in virus titer.

### Duration of different *M. p. nicotianae* biological stages on CMV infected plants under 2b silenced and non-silenced conditions

Development times of different life stages of *M. p. nicotianae *were successful on CMV infected plants under 2b silenced and non-silenced conditions, which summarizes observed biological attributes, including the development times of each life stage. Statistical analysis of the biological period showed significant differences (*P* < 0.05) between different treatments. The period length of the adult female aphid on CMV treatments (19.07 ± 0.71 days) is the highest and on CMV-2b.h treatment (11.59 ± 1.03 days) is the lowest. The longest (25.11 days) and shortest (18.06 days) female total longevity were recorded on CMV and CMV-2b.h, respectively (Table 1).

**Table 1:** Development time (days ± SE) of different stages and longevity (days ± SE) of *Myzus persicae-nicotianae* biological stages of on *Cucumber mosaic virus* (CMV) infected plants under 2b silenced and non-silenced conditions.

	Treatments							
	Healthy		CMV		CMV-PFGC5941		CMV-2b.h	
Stage	*n*	Mean ± SE	*n*	Mean ± SE	*n*	Mean ± SE	*n*	Mean ± SE
Nymph 1	44	1.89 ± 0.09 a	46	1.78 ± 0.09 a	49	1.92 ± 0.11 a	39	1.95 ± 0.08 a
Nymph 2	40	1.50 ± 0.09 a	46	1.41 ± 0.08 a	48	1.48 ± 0.11 a	37	1.41 ± 0.09 a
Nymph 3	39	1.46 ± 0.08 a	46	1.24 ± 0.07 b	47	1.34 ± 0.08 a	33	1.42 ± 0.09 ab
Nymph 4	39	1.54 ± 0.08 b	46	1.61 ± 0.07 b	45	1.51 ± 0.08 a	32	1.72 ± 0.10 a
Preadult	39	6.38 ± 0.15 ab	46	6.04 ± 0.11 a	45	6.02 ± 0.11 ab	32	6.47 ± 0.16 a
Adult (female)	39	13.72 ± 0.98 b	46	19.07 ± 0.71 a	45	16.93 ± 0.76 a	32	11.59 ± 1.03 b
Female total longevity	39	20.1 ± 1.01 c	46	25.11 ± 0.72 a	45	23.13 ± 0.78 b	32	18.06 ± 1.03 c

**Figure 1:**
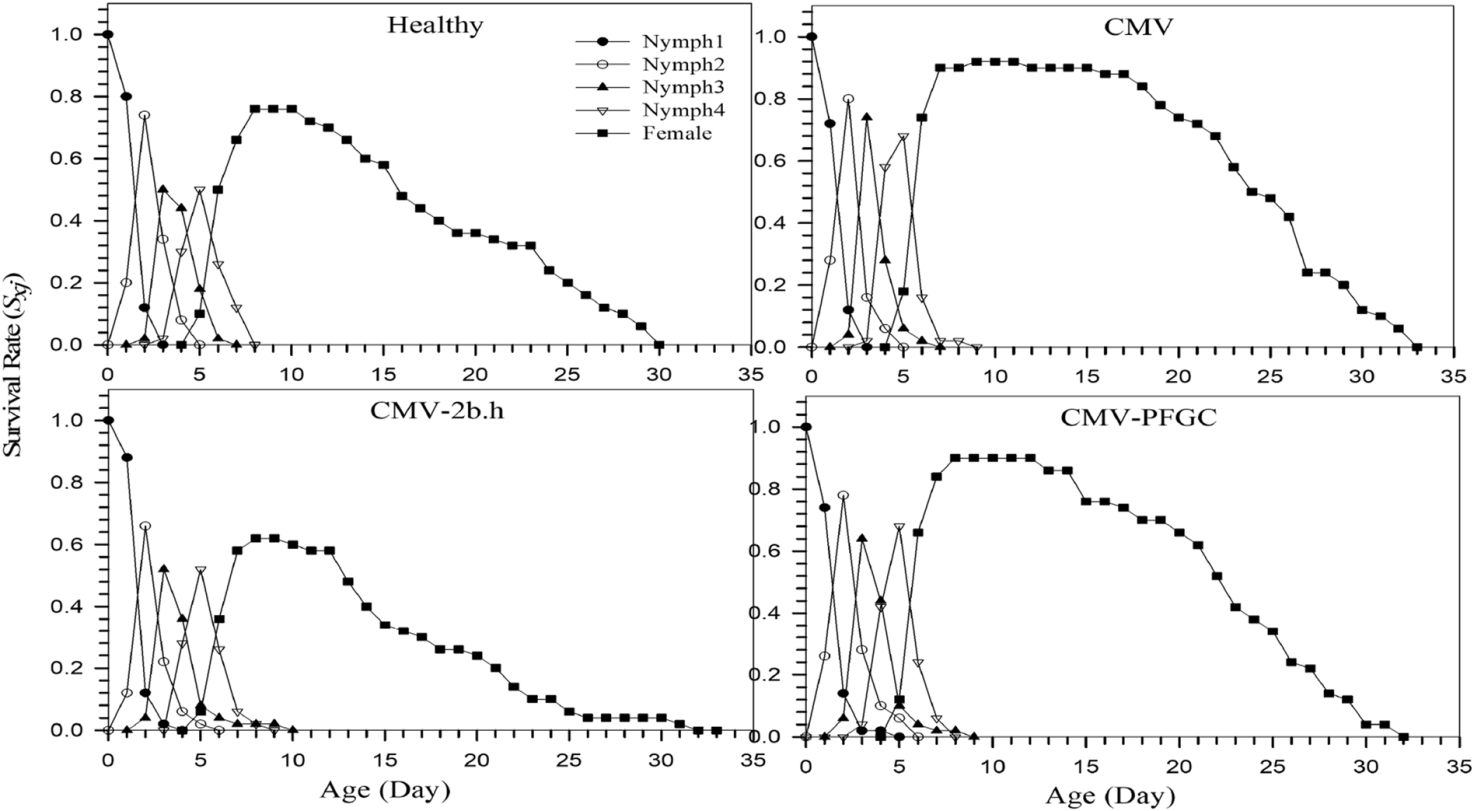
Age-stage survival rate (*s*_*xj*_) of *Myzus persicae-nicotianae* biological stages of on *Cucumber mosaic virus* (CMV) infected plants under 2b silenced and non-silenced conditions.

Age-specific survival rate (*s*_*xj*_) represents the probability of survival of newborn individuals at the *x* age and *j* growth stage. Based on the results of the age-specific survival rate of *M. p. nicotianae* under the influence of different treatments for the adult female, the highest amount related to CMV treatment (92) and the lowest amount related to CMV-2b.h treatment (62) was observed (Fig. 1).

**Figure 2:**
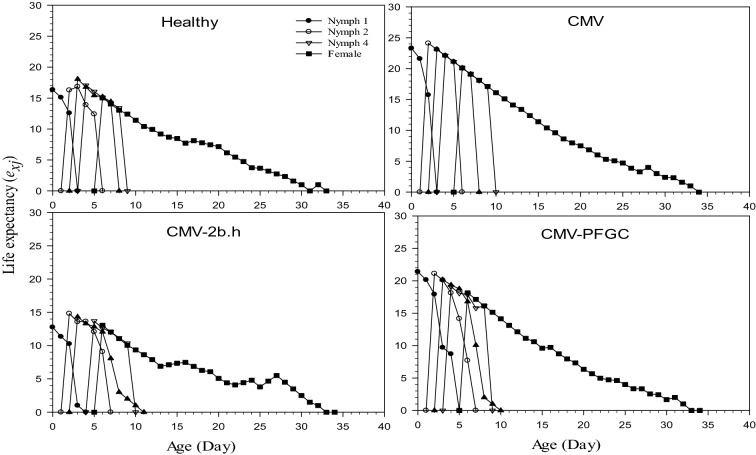
Age-stage-specific life expectancy (*e*_*xj*_) of *Myzus persicae-nicotianae* biological stages of on *Cucumber mosaic virus* (CMV) infected plants under 2b silenced and non-silenced conditions.

The age-stage-specific life expectancy (*e*_*xj*_) demonstrates the influence of the different temperatures on the expected lifespan of *M. p. nicotianae* (Fig. 2). It gives them time that individuals of age *x* and stage *j* are expected to live after age *x*. The curves of *e*_*x*_ normally decreased with age, because aging is the sole mortality factor in laboratory conditions. According to the results of *M. p. nicotianae*, the life expectancy of aphid in four different treatments, in all biological stages of aphid, CMV treatment showed the highest value, and CMV-2b.h treatment showed the lowest value (Fig. 2).

Reproduction parameters of *M. p. nicotianae* are given in Table 2. The reproduction parameters revealed that adult preoviposition (APOP) and total preoviposition period (TPOP) period of *M. p. nicotianae* females were significantly different on four different treatments. Comparison of the total pre-oviposition period for CMV-2b.h treatment (7.53 ± 0.18 days) and CMV treatment (6.83 ± 0.13 days) were the highest, and lowest amount, respectively. Also, the mean reproductive period of *M. p. nicotianae*, in CMV treatment (67.3 ± 2.35 nymph/female) was the highest and in CMV-2b.h treatment (26.26 ± 2.88 nymph/female) was the lowest. The daily reproductive, for CMV treatment (15.78 ± 0.52 days) and the CMV-2b.h treatment (8.41 ± 0.69 days) have the highest and lowest values, respectively (Table 2).

**Table 2:** Adult pre-oviposition (APOP), Total pre-oviposition period (TPOP), Reproductive period (nymph/female), and Reproductive days of *Myzus persicae-nicotianae* on *Cucumber mosaic virus* (CMV) infected plants under 2b silenced and non-silenced conditions.

Parameters	Different treatment							
	Healthy		CMV		CMV-pFGC		CMV-2b.h	
	*n*	Mean ± SE	*n*	Mean ± SE	n	Mean ± SE	*n*	Mean ± SE
APOP	39	0.95 ± 0.1 a	46	0.78 ± 0.11 a	45	0.96 ± 0.08 a	32	1.06 ± 0.12 a
TPOP	39	7.33 ± 0.13 a	46	6.83 ± 0.13 b	45	7.16 ± 0.12 ab	32	7.53 ± 0.18 a
Reproductive period (nymph/female)	39	33.31 ± 2.6 c	46	67.3 ± 2.35 a	45	47.24 ± 2.4 b	32	26.62 ± 2.88 c
Reproductive days	39	9.95 ± 0.73 c	46	15.78 ± 0.52 a	45	13.24 ± 0.55 b	32	8.41 ± 0.69 c

Standard errors were estimated by using the bootstrap technique with 100,000 resamplings. Means followed by the same letter in a row indicate no significant difference between cultivars (paired bootstrap test, *P* < 5%).

**Figure 3:**
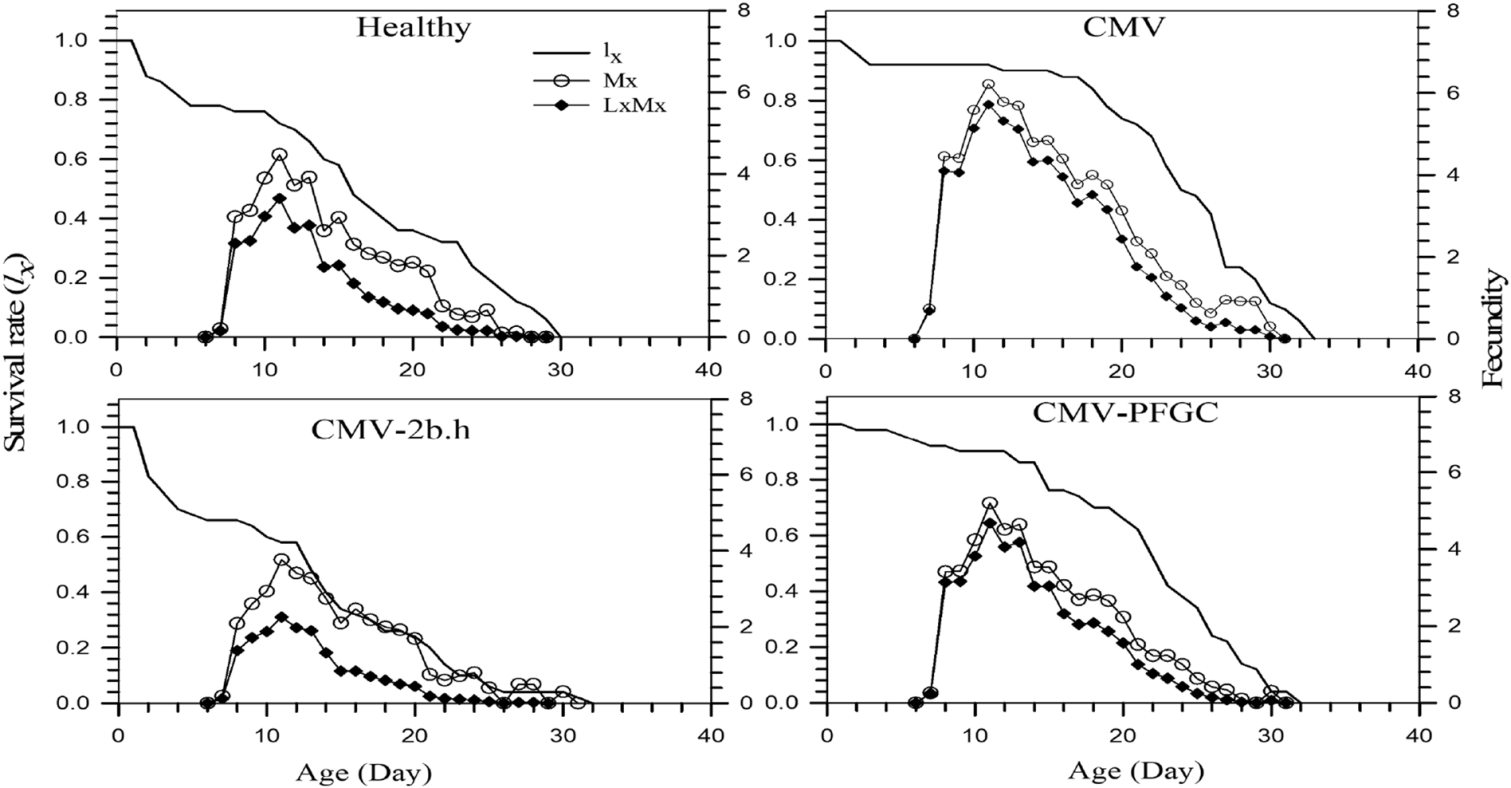
Age-specific survival rate (*l*_*x*_), age-specific fecundity (*m*_*x*_) and age-specific maternity (*l*_*x*_*m*_*x*_) of *Myzus persicae-nicotianae* on *Cucumber mosaic virus* (CMV) infected plants under 2b silenced and non-silenced conditions.

The age-specific survival rate (*l*_*x*_), fecundity (*m*_*x*_), and net maternity (*l*_*x*_*m*_*x*_) of *M. p. nicotianae* are plotted in Fig. 3. Fecundity rates (*m*_*x*_) of the aphids were greatly influenced by four treatments (Fig. 3) and the results suggested differences in age-specific infected plants under 2b silenced and non-silenced conditions dependent fecundity patterns. The reproductive period begins at age of 7th, at Healthy and CMV-2b.h, respectively. The reproductive being highest between the 10th and 20th days at all four treatments. Based on the results, the highest Reproductive period (*m*_*x*_) in the CMV treatment (6.21 nymphs on day 11) and the lowest value in the CMV-2b.h treatment (3.76 nymphs on day 11) were observed. Also, the age-specific reproductive period of the female growth-stage of female aphids (*l*_*x*_*m*_*x*_) was in CMV treatment (5.72 nymphs on day 11) and the CMV-2b.h treatment (2.26 nymphs on day 11) has the highest and lowest values (Fig. 3).

**Figure 4:**
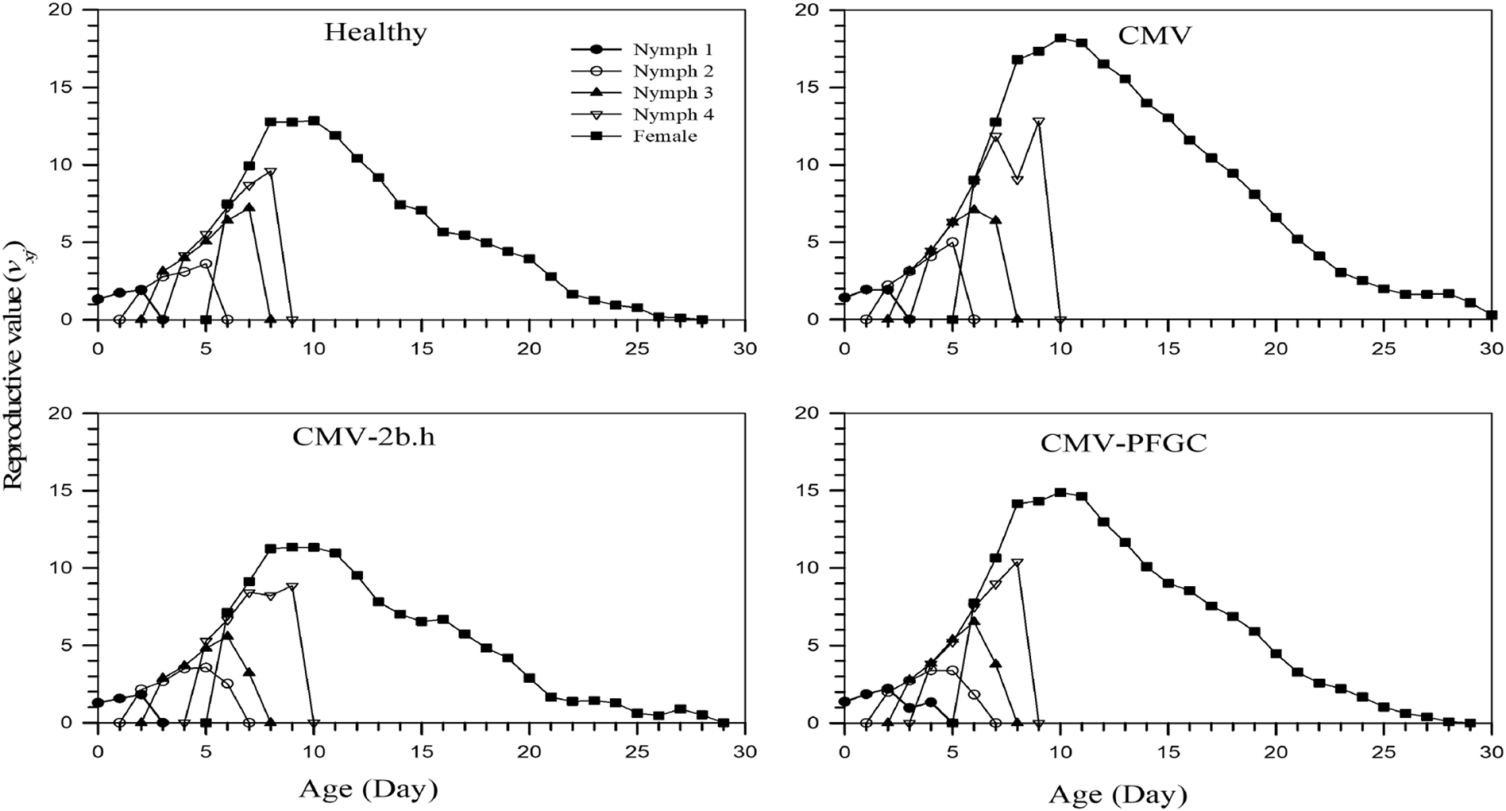
Age-stage-specific reproductive value (*v*_*xj*_) of *Myzus persicae-nicotianae* on *Cucumber mosaic virus* (CMV) infected plants under 2b silenced and non-silenced conditions.

The age-stage reproductive value (*v*_*xj*_) of *M. p. nicotianae* on CMV infected plants under 2b silenced and non-silenced conditions are presented in Fig. 4. The reproduction value (*v*_*xj*_) is the number of progeny that is expected to be produced by the female at the age of *x* and growth stage *j* for the rest of life. Among the four treatments, the *v*_*xj*_ increased significantly when adults emerged, for example, the *v*_*xj*_ of *M. p. nicotianae* on CMV jumped to 18 for 10 days female adults while on CMV-2b.h treatment jumped to 11 for 9 days female adults (Fig. 4).

The results of the paired bootstrap test showed a significant difference between the net reproductive rate (*R*_0_), intrinsic rate of increase (*r*), mean generation time (*T*), and finite rate of increase (*λ*). Table 3 summarizes the effect of the different treatments on population parameters based on the paired bootstrap. Data analysis showed a significant difference between treatments. The net reproductive rate (*R*_0_), which represents the growth rate of each generation of the population, in CMV treatment with 61.92 ± 3.353 female/generation at a higher level than CMV-pFGC, Healthy and CMV-2b.h treatment values of 42.52 ± 2.924, 25.98 ± 2.794, and 17.04 ± 2.570 female/generation, respectively (Table 3). Our results revealed that the intrinsic rate of increase (*r*) in CMV treatment (0.348 ± 0.006 day^−1^) had the highest level compared to other treatments and CMV-pFGC, Healthy and CMV-2b.h treatments were placed at lower levels with 0.319 ± 0.007, 0.285 ± 0.010, and 0.246 ± 0.014 per day, respectively (Table 3). The finite population growth rate (λ) indicates the rate of increase in the stable population each day compared to the previous day. The value of this parameter in CMV treatment (1.417 ± 0.009 per day) at the highest level and CMV-pFGC, Healthy control plants and CMV-2b.h with 1.375 ± 0.009, 1.330 ± 0.014, and 1.279 ± 0.018 per day were at lower levels, respectively (Table 3).

**Table 3:** Life table parameters (mean ± SE) *Myzus persicae-nicotianae* feed on *Cucumber mosaic virus* (CMV) infected plants under 2b silenced and non-silenced conditions.

Parameter	Different treatment			
	Healthy	CMV	CMV-pFGC	CMV $-2b.h$
*R*_0_ (offspring/individual)	25.98 ± 2.79 c	61.91 ± 3.35 a	42.51 ± 2.92 b	17.04 ± 2.57 d
*r* (day^−1^)	0.28 ± 0.01 c	0.34 ± 0.006 a	0.31 ± 0.007 b	0.24 ± 0.01 d
*λ* (day^−1^)	1.32 ± 0.01 c	1.41 ± 0.009 a	1.37 ± 0.009 b	1.27 ± 0.018 d
*T* (day)	11.42 ± 0.2 a	11.83 ± 0.16 a	11.75 ± 0.15 a	11.52 ± 0.27 a

Standard errors were estimated by using the bootstrap technique with 100.000 resampling. The difference was compared with paired bootstrap test (*P* < 0.05). The means within a row followed by different letters indicate significant differences between the six cultivars.

*R*_0_ net reproductive rate, *r* intrinsic rate of increase, *λ* finite rate of increase, *T* mean generation time.

## Discussions

Pest insects are the major limitations for food production and crop growth and their control becomes one of the most important agricultural practices around the world. Aphids have belonged the herbivorous insects that are distributed around the world and are the major crop pests that cause significant economic losses due to direct and indirect damage through feeding and virus transmission^27,28^. In nature, the control of pests and diseases depends on their interactions with plants and each other. Therefore some synergistic interactions were revealed between pests and plant diseases. Although most studies emphasize on the important role of the aphids on virus transmission, some other researches clearly showed the CMV role on the susceptibility to aphid vectors^20^. Therefore, in this study, we researched the pathogen-derived resistance to the aim of resistance to aphid vectors.

Among aphids, *M. p. nicotianae*, which is a subspecies of *M. persicae* and is particularly adaptable with tobacco, can cause significant economic loss^27,29^.

Plant-mediated RNAi has been shown to effectively inhibit the expression of target genes, and because of having the specific target, it becomes a new way to prevent and control agricultural pests^30^.

Nowadays, using of life table becomes a safe way to determine the best time to control pests, the toxicity of insecticides, and the study of pests populations^31^. The Life Table provides useful information on reproductive rates, population growth rates, and mortality rates for pest control and management^32^. The used sequence of CMV-2b in this research was aligned with the available sequences of *N. tabaccum* and *M. persicae* for checking the off-target effects. The blasting search was showed no sequence similarity of more than 18 bp to silence any sequences in these two species. However, the effect of 2b silencing construct in healthy plants on *M. persicae* was not further considered.

The data showed the highest amount of female aphids on CMV-infected treatments (25.11 days) and the lowest amount on the CMV-2b silenced treatments (CMV-2b.h) (18.06 days). Similar to the present study, Ullah et al.^33^ by RNA silencing of Chitin Synthase 1 (CHS1) gene of cotton aphid *Aphis gossypii* showed that by continuous feeding of aphids from ds-CHS1 for 72 h, the longevity of *A. gossypii* compared to control was significantly decreased (38%) (Table 1).

This finding is consistent with Ziebell et al.^20^ who showed aphids were placed on Fny-CMV infected tobacco plants exhibited incensement feeding. While, in other researches, it was shown an increment of some distasteful substances by Fny-CMV in squash and Arabidopsis thaliana plants inhibit lengthened feeding on the phloem^34,35^. Although CMV infected squash plants are considered as a poor host for aphids, the increment of volatile emissions attracted aphids^36,37^. This may enhance the virus acquisition from infected hosts and causes rapid virus dissemination^10^. Shi et al.,^26^ indicated that the number of aphids on infected plants increased significantly at the beginning of infection but also decreased significantly after the virus titer reached a certain level. They fined the number of winged aphids increased on CMV-infected plants from day 9, and the highest number was on day 15th^26^. Therefore, increment the number of winged aphids cause rapid virus dissemination.

Obtained data from this research showed the highest survival duration of adult female aphids on CMV infected plants, while the survival duration for 2b silenced (CMV-2b.h) was the lowest (Table 1 and Fig. 1). Thus, our results show that the silencing of 2b causes a reduction in longevity of female aphids on CMV infected plants (Table 1). Other researches revealed while the number of aphids on CMV infected and mock-inoculated are same at 3 dpi, but it increased significantly from the day 6th to 15th on CMV treatments and it is related to proteinase inhibitor, which is reduced from the day 3-15th dpi, and it shows time-dependent of aphid performance on CMV-infected plants^26^. Expression of proteinase inhibitors induces by the induction of jasmonic acid^38,39^**.** At the early stage of infection, CMV-infected plants show a low level of plant defense, therefore, the aphid population increases rapidly. But when the viral titer reaches the stable level in plants, plant defense reaches a higher level^40^. As Figs. 3 and 4 show, the aphid survival and reproduction increased on CMV infected plants and this result is in line with Ziebell et al.,^20^ as reported the increment of feeding on the phloem, survival and reproductive of aphids on CMV infected plants. The survival of *M. persicae* was increased on tobacco plants infected with CMV-Fny, but decreased on the tobacco plant infected by CMV-Fny 2b mutated (CMVΔ2b)^41^. Moreover, in our research, silencing of CMV-2b caused the reduction of aphids survival and reproduction, compared to the other treatments, even healthy plants (Fig. 3 and Table 2), and this is an interesting question, why CMV-2b silenced treatments shows more reduction of aphid survival and reproduction? One possibility is, that infiltration of leaves by agrobacterium cell suspension causes the induction of salicylic acid (SA) and jasmonic acid (JA) pathway. SA and JA have been reported as effective phytohormones against aphids and whiteflies^26,42,43^. SA and JA synergistic and antagonistic interactions change during plant-CMV-aphid interactions^44^, and also other microbes can influence this interaction. However, the increment of resistance in plants expressing empty vector maybe because of the changes in these phytohormones after infiltration by agrobacterium. The CMV- 2b protein targets the NPR1, an important defensive related protein^24,25^, and the expression of SA relative genes was small. While at the early stages of CMV infection, the expression of OPR3 at the upstream of JA and the repressor of NPR1, was induced. But on the 9th day, when the viral titer reaches the highest peak, the induction of SA relative genes such as NPR1 was at the highest level, and the expression of genes downstream of JA was suppressed^26^.

Analysis of life expectancy of aphids on different treatments in the present experiment indicated the highest value on CMV treatment and the lowest value on CMV-2b silenced (CMV-2b.h) and it showed CMV infected plants are more desirable for aphids feeding (Fig. 2). Our founding showed the highest pre-nymph period on CMV-2b.h treatment and the lowest on CMV-infected plants. The highest value of reproduction period and the highest number of daily nymph were observed on CMV-infected plants, although the lowest was for the CMV-2b.h treatment. Transgenic potatoes expressing dsRNAs against Colorado potato beetle *Leptinotarsa decemlineata* juvenile hormone indicated dramatically decrement of the *L. decemlineata* JHAMT transcription and negative effect on the egg-laying, growth, and development of the target pest^45^.

In the present study, the highest reproductive period was obtained for the CMV-infected treatment (67.3 nymph/female) and the lowest value was for CMV-2b.h treatment (26.62 nymph/female) (Table 2). Therefore, silencing of CMV-2b in CMV infected plants causes’ reduction of the reproductive period. Wuriyanghan and Falk^46^, examined the induction of RNAi using Tobacco mosaic virus (TMV) as a vector against Actin and ATPase of potato psyllid *Bactericera cockerelli*, and observed no differences between control and treatment plants 7 days after feeding on different treatments, while significant difference was observed for nymph number after 14 days, and it was decreased by ~ 38% for TMV-ATPase and ~ 40% for Actin silenced. They also showed these two dsRNAs decreased psyllid reproduction.

Daily nymphs’ number was the highest on CMV-infected plants (15.78), while it was the lowest value for CMV-2b.h (8.41). These data suggest increasing aphid reproduction by CMV. While silencing of CMV-2b reduces the nymph number of aphid vectors. Therefore, these results indicated the coexistence of CMV and aphid vectors during the evolution. Therefore, during this co-evolution, both benefit each other, as suggested by other researchers^20,26^. The reproductive value was higher for CMV-infected plants in comparison with the other treatments. Silencing of CYP392A4 against *Tetranychus* cinnabarinus showed a decrement of daily eggs from 69.38 for wild cotton plants to 46.04 for transgenic cotton expressing CYP392A4 dsRNA and the total laying period of *T. cinnabarinus* on transgenic plants expressing dsRNA was consistently lower than the wild cotton plants^47^.

Our results showed that reproduction and life table parameters are important parameters for assessing *M. persicae-nicotianae* feed on Cucumber mosaic virus (CMV) CMV infected plants under 2b silenced and non-silenced conditions. The intrinsic rate of the natural increase reflects several biological parameters such as survival, development, and fecundity and is the best indicator for the physiological attributes of an insect regarding its capability to increase; therefore, it can be used to forecast the prospective severity of the insect pests and good bioclimatic index for comprehensive susceptibility of a host plant to phytophagous arthropods. The higher value of *r* for insects on the plants shows more sensitivity to insect nutrition and the less value manifests more resistant^48^. The results of this research for population growth parameters was shown the highest intrinsic rate of birth and mortality for CMV infected plants (0.348), while this parameter was the lowest (0.246) for CMV-2b.h (Table 3) and this means the silencing of 2b gene causes reduction of the intrinsic rate of population growth. Therefore, the CMV-infected plants can be considered as a sensitive treatment and CMV-2b.h treatment as an aphid-resistant. The higher intrinsic rate of population growth (*r*) was for CMV-infected plants due to a high reproduction period, lower mortality, and shorter growth duration before maturity. The finite population growth rate (*λ*) showed the same results as the intrinsic rate of population growth, and it was 1.417 per day for CMV infected plants, while it was 1.279 per day for CMV-2b.h. However, the results of population growth parameters showed the silencing of CMV-2b in CMV infected plants causes induction of resistance against aphids (Fig. 4 and Table 3).

## Conclusions

Evaluation of intrinsic population growth rate, which indicates the resistance evaluation index, on tobacco plants expression 2b silencing gene construct and empty vector as a control, indicated that tobacco plants infected with CMV are more sensitive to *M. p. nicotianae* in comparison with the healthy plants*.* Our finding showed CMV infected plants expressing 2b silencing construct, represented more resistance to *M. p. nicotianae* compare to the CMV infected plants expressing empty vector. Also, due to the higher intrinsic rate of population growth (*r*) and the finite population growth rate (*λ*) for CMV-infected plants, which lead to more aphids reproducing, their accumulation and damage, and the CMV-infected plant is known to be a more desirable host.-In conclusion, the collected data of this research reflected the induction of resistance to both CMV and aphid vectors by silencing of CMV-2b. Therefore, based on the results, it can be hoped to achieve the resistance to pests and pathogens at the same time, by silencing one gene and it can lead to the reduction of chemical pesticide application.

## Materials and methods

### Plant, virus, plasmids and virus inoculation

Commercial tobacco (*Nicotiana tabacum*) seeds (Cultivar Samsun) were obtained from The University of Tarbiat modares and planted in 1.5 L plastic pots containing potting mix 1:1:1 sterilized soil, sand, and manure. During the experiments, the plants were kept in a greenhouse at 25 ± 2 °C. The two cotyledonary leaves of seedlings at the 3-to-4 leaf stage were mechanically inoculated to the CMV-Kurdistan isolate (MK796159) using carborundum powder and phosphate buffer (0.1 M K_2_HPO_4_, 0.15% 2-β mercaptoethanol and pH = 8.5)^49^. Control plants (Mock plants) were rub inoculated using phosphate buffer alone. Plants were used for the experiment at 10 days post-inoculation (dpi). The recombinant plasmid for silencing of CMV-2b (pFGC-C.h) was provided from the department of plant pathology, Tarbiat Modares University^50^. The CMV-2b silencing construct was contained 145 bp of CMV-2b (2442–2586 nt of S72187.1). The CMV-2b gene fragment was first cloned into the Kannibal plasmid as a hairpin structure containing *pdk* intron between sense and antisense orientation. Then the hairpin structure was as sub-cloned into pFGC5941 under 35S promoter.

### ELISA

Indirect ELISA was performed for detection of CMV infected plants and plant resistant assay to CMV. For this aim, samples were collected from plants at 15 dpi and they were checked using CMV polyclonal antiserum produced by the Plant Virus research Centre (Shiraz University, Iran) with 1/300 dilution and the Goat anti Rabbit alkaline phosphatase (Promega, USA) with 1/7500 dilution. Absorption was checked at 405 nm and samples with the twofold absorption compare to the control plants, described as infected plants. Plant samples were used as healthy and infected plants were checked using the ELISA test before the performance of the detached leaf assay.

### Raising aphids

Wingless (apterous) *Myzuses p. nicotianae* individuals were originally collected from tobacco fields in Marivan, Iran, and morphologically identified by the University of Kurdistan entomologists. Tobacco aphids from the field were reared on tobacco (*N. tabacum*, Samsun Cultivar) plants in a greenhouse set at 25 ± 1 °C and 75 ± 5% RH. The plants that aphids were fed on were checked for CMV infection using RT-PCR and indirect ELISA test. Aphids were raised on tobacco plants to reach the desired population needs for the experiment.

### Agrobacterium transformation

The 2b silencing plasmid (pFGC-C.h) was transformed to the Agrobacterium LBA 4404 strain by heat shock method^51^. Hence, the agrobacterium was cultured in a shaker incubator at 28 °C for overnight and the competent cell was prepared by a protocol described^51^. The tubes containing 100 µl of Agrobacterium competent cells were placed in a water bath for 5 min at 37 °C, then added 800 µl of LB medium and placed on a shaker incubator at 28 °C for 2 h. After that, 200 µl of the agrobacterium cell suspension was overnight cultured in an incubator at 28 °C on the LBA medium containing rifampicin and kanamycin.

### Transient expression of the gene construct

Agrobacterium clones containing the gene construct were cultured for two days in a liquid YEB medium containing kanamycin and rifampicin on a shaker incubator (150 rpm, 28–30 °C). Thereupon, the cell suspension was precipitated by centrifugation at 5,000 rpm for 5 min, the supernatant phase was removed and the bacterial cell pellet was resuspended in sterile distilled water to OD_600_ = 0.6. Therefore, the cell suspension was injected under two leaves (third and fourth leaves) of each tobacco plantlet using a syringe. After injection for expression of small RNAs, plants were kept for 4 days under greenhouse conditions (25 ± 2 Cº) before the inoculation by CMV. Transient expression was carried out using agrobacterium cell suspension containing pFGC-C.h for the treatment and cell suspension harboring empty vector as a control (pFGC5941),

### RNA extraction

RNA was extracted using lithium chloride (Yu et al. 2012). For RNA extraction plant leaves powdered using liquid nitrogen, then 0.1 gr of leaves powder transferred to 1.5 ml microtube and 700 µl of extraction buffer (3% CTAB_3_, 100 mM Tris–HCl pH 8.0, 1.4 M NaCl, 20 mM EDTA, 5%PVP, 1%) were added. After that, the tubes were vortexed vigorously for about 1 min, kept at 55 ºC for 10 min, added 400 of chloroform, vortexed for about 20S, and then centrifuged (10 min, 12,000 rpm). Then, the supernatant was transferred to a new tube, added ½ vol of lithium chloride (10 M), and then samples were kept overnight at 4 ºC. Then, the samples were centrifuged in the same condition and after that, the supernatant was removed, the pellet was washed using 70% ethanol and then the RNA pellet was re-suspended in 100 µl of DEPC water.

### RT-PCR

cDNA was synthesized using random hexamer primer and HyperscriptTM RT Mastermix (GeneAll, Korea) using the recommended protocol. For this aim, 2 µl (50 ng/µl) of RNA was used in 10 µl of RT reaction. The polymerase chain reaction was carried out using 3 µl of cDNA and 10 ng of CMV-F: *GTAGACATCTGTGACGCGA* and CMV-R: *GCGCGAAACAAGCTTCTTATC* primers^52^ and Ampliqon (Denmark) PCR master mixed. The temperature cycle was 94ºC for 2 min, and 35 cycles of 94ºC for 35 S, 54ºC for 35 S, and 72 ºC for 90 S, and the final extension was 72 ºC for 5 min.

### Evaluation of *M. p. nicotianae* demographic parameters on tobacco plant treatments

To determine life table parameters of tobacco aphids on four different tobacco treatments in laboratory conditions, at 25 ± 2 °C, 60 ± 5 relative humidity, and 8:16 photoperiod were investigated. *M. p. nicotianae* was transferred separately and homogeneous nymphs (24 h) were collected from the leaves and used for testing. In this study, 50 first-age aphids’ nymphs were separated on tobacco leaves (leaves of 5–8 of tobacco plants) in Petri dishes (50 first-age nymphs on 50 tobacco leaves in Petri dishes (Fig. S1).

The circumference of the leaves was covered with cotton to prevent aphids from escaping and the cotton was soaked daily to maintain the moisture inside the Petri dishes. Petri dishes that aphids had become adult were visited every 24 h. We have conducted the period duration, the rate of development, and survivorship of nymphs until the end of adult aphids' life, in an environmental chamber. The number of progeny by each adult female was monitored and recorded daily. To calculate population growth parameters, the data were adjusted and calculated based on the female age (*x*) and the survival rate of adult females at the age of x (*l*_*x*_), and the number of female progeny produced at the age of x (*m*_*x*_).

### Life history parameters

The life history raw data of all individuals was analyzed based on the age-stage, two-sex life table^53,54^. The life table parameters, including the age-stage specific survival rate (*s*_*xj*_) (where *x* = age in days and *j* = stage); the age-stage-specific fecundity (*f*_*x*_) (daily number of eggs produced per female of age *x*); the age-specific survival rate (*l*_*x*_); the age-specific fecundity (*m*_*x*_), adult preoviposition period (APOP, the preoviposition period counted from the adult emergence), total preoviposition period (TPOP, the preoviposition period counted from birth) and the population growth parameters, the net reproductive rate (*R*_0_), intrinsic rate of increase (*r*), finite rate of increase (*λ*), mean generation time (*T*) and were calculated accordingly. According to Chi and Liu^54^, the net reproductive rate is defined as the mean number of offspring that an individual can produce during its lifetime. The above-mentioned parameters were calculated as:

1 $$l_{x} = \sum\limits_{j = 1}^{k} {s_{xj} }$$

where *k* is the number of stages. The age-specific fecundity (*m*_*x*_) was calculated as:

2 $$m_{x} = \frac{{\sum\nolimits_{j = 1}^{k} {s_{xj} f_{xj} } }}{{\sum\nolimits_{j = 1}^{k} {s_{xj} } }}$$

The net reproduction rate is defined as the mean number of offspring that an individual can produce during its lifetime and was calculated as:

3 $$R_{0} = \sum\limits_{x = 0}^{\omega } {l_{x} m_{x} }$$

The intrinsic rate of increase was estimated from the Euler-Lotka formula using the method of iterative bisection with the age indexed from 0^55^ as:

4 $$\sum\limits_{x = 0}^{\omega } {e^{{ - r\left( {x + 1} \right)}} l_{x} m_{x} = 1}$$

The finite rate (*λ*) was calculated as:

5 $$\lambda = e^{r}$$

The mean generation time is the time length that a population needs to increase to *R*_0_-fold of its size as the population reaches the stable age-stage distribution and was calculated as:

6 $$T = \frac{{\ln R_{0} }}{r}$$

The standard errors of the developmental times, fecundity, reproduction period, and population parameters were estimated using the bootstrap method^56,56^, with 100,000 bootstraps to obtain stable estimates of standard errors^57^. The paired bootstrap test was used to compare differences among the treatments^56^. The computer program TWOSEX-MSChart^58^ was used for the raw data analysis and calculation of population parameters. All figures were plotted using Sigmaplot 12.3 software.

#### Population projection

We used the method described by^59^ and the computer program TIMING^58^ to project the population growth. To show the variability of population growth, we sorted the 100,000 bootstrap results of the finite rate (*λ*) to find the 2.5th and 97.5th percentiles, i.e., the 2,500th and 97,500th sorted bootstrap samples. We then used the bootstrap life table samples that generated the 2.5th and 97.5th percentiles finite rate of increase (λ) to project the population. The results represent the confidence interval of the projected population growth^60^. We used the method devised by Chi 1990 included in the computer program TIMING- MSChart^58^ for hypothetical population projection based on life table parameters.

#### A statement on guidelines

Plants and leave sampling in our study complies with the Iranian National plant Gene Bank guideline and legislation.

## Supplementary Information

Supplementary Information.

## References

[1] Garcı´a-Arenal, F. and Palukaitis, P. García-arenal: desk encyclopedia of plant and fungal... Google Scholar. *Acad. Press* 614 (2008).

[2] Zhou B, Wang F, Zhang X, Zhang L, Lin H (2017). Sequencing and phylogenetic analysis of tobacco virus 2, a polerovirus from Nicotiana tabacum. Arch. Virol..

[3] Hily JM (2014). The Relationship between Host Lifespan and Pathogen Reservoir Potential: An Analysis in the System Arabidopsis thaliana-Cucumber mosaic virus. PLOS Pathog..

[4] Gildow F (2008). Transmission efficiency of Cucumber mosaic virus by aphids associated with virus epidemics in snap bean. Phytopathology.

[5] Yang Y, Kim KS, Anderson EJ (2007). Seed transmission of cucumber mosaic virus in spinach. Phytopathology.

[6] Nalam V, Louis J, Shah J (2019). Plant defense against aphids, the pest extraordinaire. Plant Sci..

[7] Jacquemond M (2012). Cucumber mosaic virus. Adv. Virus Res..

[8] Yoon J-Y, Palukaitis P, Choi S-K (2019). CHAPTER 1: host range. Cucumber Mosaic Virus.

[9] Groen S, Wamonje F, Murphy A, Carr J (2017). Engineering resistance to virus transmission. Curr. Opin. Virol..

[10] Donnelly R, Cunniffe NJ, Carr JP, Gilligan CA (2019). Pathogenic modification of plants enhances long-distance dispersal of nonpersistently transmitted viruses to new hosts. Ecology.

[11] Blanc S, Michalakis Y (2016). Manipulation of hosts and vectors by plant viruses and impact of the environment. Curr. Opin. Insect Sci..

[12] Saurav GK, Rana VS, Popli S, Daimei G, Rajagopal R (2019). A thioredoxin-like protein of Bemisia tabaci interacts with coat protein of begomoviruses. Virus Genes.

[13] Carmo-Sousa M, Moreno A, Garzo E, Fereres A (2014). A non-persistently transmitted-virus induces a pull-push strategy in its aphid vector to optimize transmission and spread. Virus Res..

[14] Brunt, A. Plant Viruses Online: Descriptions and Lists from the VIDE Database. http://biology.anu.edu.au/Groups/MES/vide/ (1996).

[15] Harris, K. F. An ingestion-egestion hypothesis of noncirculative virus transmission. *Aphids Virus Vectors* 165–220 (1977).

[16] Lax C (2020). The Evolutionary Significance of RNAi in the Fungal Kingdom. Int. J. Mol. Sci..

[17] Rodrigues TB, Figueira A (2016). Management of Insect Pest by RNAi — A New Tool for Crop Protection. RNA Interf..

[18] Mathioudakis MM (2021). Molecular characterization of the coat protein gene of greek apple stem pitting virus isolates: Evolution through deletions, insertions, and recombination events. Plants.

[19] Zhang X (2006). Cucumber mosaic virus-encoded 2b suppressor inhibits Arabidopsis Argonaute1 cleavage activity to counter plant defense. Genes Dev..

[20] Ziebell H (2011). Cucumber mosaic virus and its 2b RNA silencing suppressor modify plant-aphid interactions in tobacco. Sci. Rep..

[21] Lai Z, Wang F, Zheng Z, Fan B, Chen Z (2011). A critical role of autophagy in plant resistance to necrotrophic fungal pathogens. Plant J..

[22] Westwood JH (2014). Interference with jasmonic acid-regulated gene expression is a general property of viral suppressors of RNA silencing but only partly explains virus-induced changes in plant–aphid interactions. J. Gen. Virol..

[23] Tungadi T (2020). Cucumber mosaic virus 2b proteins inhibit virus-induced aphid resistance in tobacco. Mol. Plant Pathol..

[24] Lewsey MG (2010). Disruption of two defensive signaling pathways by a viral RNA silencing suppressor. Mol. Plant-Microbe Interact..

[25] Love AJ (2012). Cauliflower mosaic virus protein P6 inhibits signaling responses to salicylic acid and regulates innate immunity. PLoS ONE.

[26] Shi X (2016). Aphid performance changes with plant defense mediated by Cucumber mosaic virus titer. Virol. J..

[27] Blackman, R. L. & Eastop, V. F. Aphids on the World’s Crops: An Identification and Information Guide, 2nd Edition - R. L. Blackman, V. F. Eastop. 476 (2000).

[28] Moury B, Fabre F, Senoussi R (2007). Estimation of the number of virus particles transmitted by an insect vector. Proc. Natl. Acad. Sci. U. S. A..

[29] van-Emden, H. & Harrington, R. Aphids as Crop Pests - CABI.org. *CABI* https://www.cabi.org/bookshop/book/9781780647098/ (2007).

[30] Yu X (2016). RNAi-mediated plant protection against aphids. Pest Manag. Sci..

[31] Ann K. Sakai *et al.* The population biology of invasive species. **32**, 305–332 10.1146/annurev.ecolsys.32.081501.11403732,305-332 (2001).

[32] Kakde AM, Patel KG, Tayade S (2014). Role of life table in insect pest management-a review. IOSR J. Agric. Vet. Sci..

[33] Ullah F (2020). RNAi-Mediated Knockdown of Chitin Synthase 1 (CHS1) Gene Causes Mortality and Decreased Longevity and Fecundity in Aphis gossypii. Insects.

[34] Mauck KE, De Moraes CM, Mescher MC (2010). Deceptive chemical signals induced by a plant virus attract insect vectors to inferior hosts. Proc. Natl. Acad. Sci..

[35] Westwood, J. *et al.* A trio of viral proteins tunes aphid-plant interactions in Arabidopsis thaliana. *PLoS One* **8**, (2013). 10.1371/journal.pone.0083066 PMC3859657 24349433

[36] Mauck KE, Smyers E, De Moraes CM, Mescher MC (2015). Virus infection influences host plant interactions with non-vector herbivores and predators. Funct. Ecol..

[37] Mauck K, De Moraes C, Mescher M (2014). Biochemical and physiological mechanisms underlying effects of Cucumber mosaic virus on host-plant traits that mediate transmission by aphid vectors. Plant. Cell Environ..

[38] Turner JG, Ellis C, Devoto A (2002). The Jasmonate Signal Pathway. Plant Cell.

[39] Zhang H, Xie X, Xu Y, Wu N (2004). Isolation and functional assessment of a tomato proteinase inhibitor II gene. Plant Physiol. Biochem. PPB.

[40] Pegadaraju V, Knepper C, Reese J, Shah J (2005). Premature leaf senescence modulated by the arabidopsis PHYTOALEXIN DEFICIENT4 gene is associated with defense against the phloem-feeding green peach aphid. Plant Physiol..

[41] Tungadi T (2017). Cucumber mosaic virus and its 2b protein alter emission of host volatile organic compounds but not aphid vector settling in tobacco. Virol. J..

[42] Shi X (2013). Plant virus differentially alters the plant’s defense response to its closely related vectors. PLoS ONE.

[43] Donovan MP, Nabity PD, DeLucia EH (2013). Salicylic acid-mediated reductions in yield in Nicotiana attenuata challenged by aphid herbivory. Arthropod-Plant Interact..

[44] Koornneef A, Pieterse C (2008). Cross talk in defense signaling. Plant Physiol..

[45] Guo W (2018). Double-Stranded RNAs High-Efficiently Protect Transgenic Potato from Leptinotarsa decemlineata by Disrupting Juvenile Hormone Biosynthesis. J. Agric. Food Chem..

[46] Wuriyanghan H, Falk BW (2013). RNA Interference towards the Potato Psyllid, Bactericera cockerelli, Is Induced in Plants Infected with Recombinant Tobacco mosaic virus (TMV). PLoS ONE.

[47] Shen G-M (2017). Transgenic cotton expressing CYP392A4 double-stranded RNA decreases the reproductive ability of Tetranychus cinnabarinus. Insect Sci..

[48] Carey, J. R. *Applied Demography for Biologists with Special Emphasis on Insects*. (Oxford University Press, Oxford, 1993).

[49] Yarwood CE (1952). The phosphate effect in plant virus inoculations. Phytopathology.

[50] Azizi A, Verchot J, Moieni A, Shams-bakhsh M (2020). Efficient silencing gene construct for resistance to multiple common bean (Phaseolus vulgaris L.) viruses. 3 Biotech.

[51] Höfgen R, Willmitzer L (1988). Storage of competent cells for Agrobacterium transformation. Nucl. Acids Res..

[52] De Blas C, Borja MJ, Saiz M, Romero J (1994). Broad spectrum detection of cucumber mosaic virus (CMV) using the polymerase chain reaction. J. Phytopathol..

[53] Chi H (1988). Life-table analysis incorporating both sexes and variable development rates among individuals. Environ. Entomol..

[54] Chi H, Liu H (1985). Two new methods for this study of insect population ecology. Bull. Inst. Zool.

[55] Goodman D (1982). Optimal life histories, optimal notation, and the value of reproductive value on JSTOR. Am. Nat..

[56] Efron, B. & Tibshirani, R. An Introduction to the Bootstrap. 436 (1994).

[57] Akca I, Ayvaz T, Yazici E, Smith CL, Chi H (2015). Demography and population projection of aphis fabae (hemiptera: aphididae): with additional comments on life table research criteria. J. Econ. Entomol..

[58] Chi H (2020). Age-stage, two-sex life table: an introduction to theory, data analysis, and application. Entomol. Gen..

[59] Chi H (1990). Timing of control based on the stage structure of pest populations: a simulation approach. J. Econ. Entomol..

[60] Huang H, Chi H, Smith C (2018). Linking demography and consumption of henosepilachna vigintioctopunctata (coleoptera: coccinellidae) fed on solanum photeinocarpum (solanales: solanaceae): with a new method to project the uncertainty of population growth and consumption. J. Econ. Entomol..

