# Supplementary material for: Induction of resistance to Myzus persicae-nicotianae in Cucumber mosaic virus infected tobacco plants using silencing of CMV-2b gene
Source: Sci Rep. 2022 Mar 8;12:4096. doi: 10.1038/s41598-022-08202-3 (PMC8904847; doi:10.1038/s41598-022-08202-3)
Supplement: Supplementary file 1 — Supplementary Information. [file 41598_2022_8202_MOESM1_ESM.docx]

**Supplementary Figures**


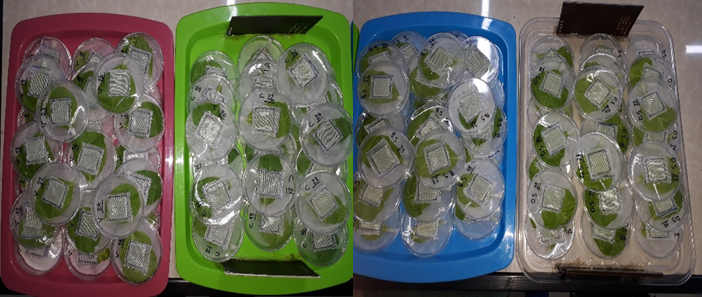


**Fig S1)** Detached leaf assay for evaluation of *M. nicotianae* demographics parameters in different treatments.

**Fig S2**) Evaluation of CMV concentration in different treatments using Indirect ELISA at 405 nm. Columns shows mean absorption of three replicates. CMV-pFGC5941) CMV infected plants expressing control plasmid (pFGC5941) CMV-pFGC-c.h) CMV infected plants expressing 2b silencing construct CMV) CMV infected plants (Positive control) H) Healthy tobacco plants (Nagative control).


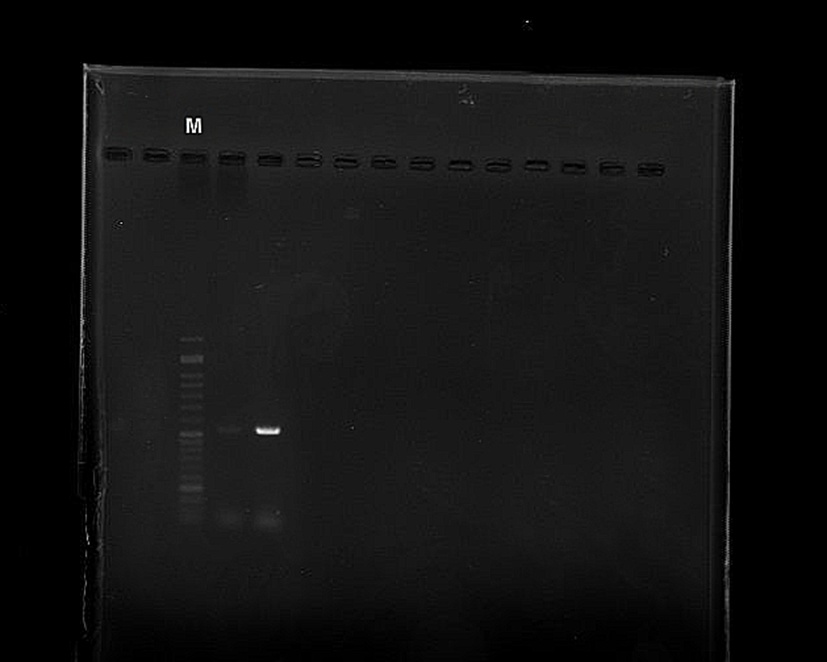


M

1

2

500 bp

**Fig S3)** PCR detection of CMV in inoculated tobacco plants. A 520 bp amplified band shows CMV infected plants. M) 100 bp Marker 1 and 2) CMV infected plants
